# The Role of Periostin in Angiogenesis and Lymphangiogenesis in Tumors

**DOI:** 10.3390/cancers14174225

**Published:** 2022-08-30

**Authors:** Adrian Wasik, Katarzyna Ratajczak-Wielgomas, Arkadiusz Badzinski, Piotr Dziegiel, Marzenna Podhorska-Okolow

**Affiliations:** 1 Division of Histology and Embryology, Department of Human Morphology and Embryology, Wroclaw Medical University, 50-368 Wroclaw, Poland; 2 Silesian Nanomicroscopy Center, Silesia LabMed: Research and Implementation Center, Medical University of Silesia, 41-800 Zabrze, Poland; 3 Department of Human Biology, Wroclaw University of Health and Sport Sciences, 51-612 Wroclaw, Poland; 4 Department of Ultrastructural Research, Wroclaw Medical University, 50-368 Wroclaw, Poland

**Keywords:** periostin, angiogenesis, lymphangiogenesis, vessels, metastases, epithelial-mesenchymal transition

## Abstract

**Simple Summary:**

Cancers are common diseases that affect people of all ages worldwide. For this reason, continuous attempts are being made to improve current therapeutic options. The formation of metastases significantly decreases patient survival. Therefore, understanding the mechanisms that are involved in this process seems to be crucial for effective cancer therapy. Cancer dissemination occurs mainly through blood and lymphatic vessels. As a result, many scientists have conducted a number of studies on the formation of new vessels. Many studies have shown that proangiogenic factors and the extracellular matrix protein, i.e., periostin, may be important in tumor angio- and lymphangiogenesis, thus contributing to metastasis formation and worsening of the prognosis.

**Abstract:**

Periostin (POSTN) is a protein that is part of the extracellular matrix (ECM) and which significantly affects the control of intracellular signaling pathways (PI3K-AKT, FAK) through binding integrin receptors (αvβ3, αvβ5, α6β4). In addition, increased POSTN expression enhances the expression of VEGF family growth factors and promotes Erk phosphorylation. As a result, this glycoprotein controls the Erk/VEGF pathway. Therefore, it plays a crucial role in the formation of new blood and lymphatic vessels, which may be significant in the process of metastasis. Moreover, POSTN is involved in the proliferation, progression, migration and epithelial-mesenchymal transition (EMT) of tumor cells. Its increased expression has been detected in many cancers, including breast cancer, ovarian cancer, non-small cell lung carcinoma and glioblastoma. Many studies have shown that this protein may be an independent prognostic and predictive factor in many cancers, which may influence the choice of optimal therapy.

## 1. Introduction

**Figure 1:**
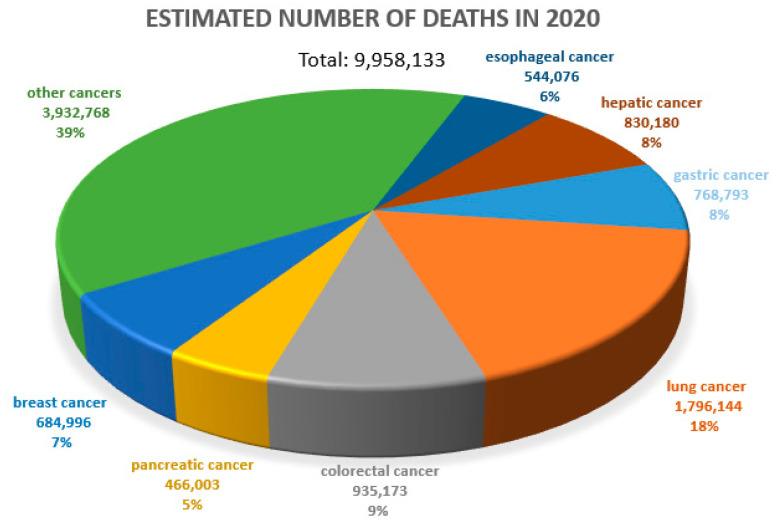
Estimated number of deaths due to all cancers worldwide. Both sexes, all ages. WHO data (2020), URL address [4], modified.

Cancer diseases are one of the most important causes of premature deaths worldwide. According to the WHO, approximately 10 million people died due to cancer in 2020 [1] (Figure 1). The prognosis in many cancers is unfavorable. Therefore, the search for prognostic and predictive markers and related new therapeutic options is so crucial. In a negative prognosis, the process of new blood vessel formation (angiogenesis), which clearly determines tumor cell metastasis, is of crucial importance [2,3].

Angiogenesis is a multistep process involving the formation of new blood vessels from the pre-existing ones [5,6]. It involves the elongation and branching of vessels in response to stimulation by proangiogenic factors [7]. Angiogenesis occurs physiologically and pathologically (e.g., in neoplastic disease). Based on many in vitro and in vivo studies, scientists have identified factors involved in the neovascularization process and its consecutive stages [2,8], which include activation of endothelial cells (EC), dilation of parent vessel wall, degradation of the basement membrane and the extracellular matrix (ECM). This is followed by EC migration towards angiogenesis stimulators and their proliferation. The next stage is related to the formation of the lumen of a new vessel and its maturation. 

The stimulation of angiogenesis may be initiated by both physical (intensive muscle work [9]), and chemical factors (e.g., integrins, prostaglandins, growth factors [10]). By binding to the receptors (VEGFR, FGFR) located on the surface of ECs, the vascular endothelial growth factor (VEGF) and the fibroblast growth factor (FGF) play an important role in the first stage of angiogenesis by initiating this process [8]. Next, proteolytic enzymes (metalloproteinases; MMPs) are activated. They are responsible for the degradation of the basement membrane and ECM, which releases proangiogenic factors in the stroma and allows EC migration [11]. Moreover, integrins (αvβ3, α2v) located on the surface of ECs interact with specific ECM components (i.e., fibronectin, laminin), which also facilitates their migration [12]. The penultimate stage involves EC proliferation, vessel lumen formation and capillary loop formation [11]. This is completed by maturation of ECs and stabilization of the vessel by the formation of the basement membrane and recruitment of adventitial cells [2].

Lymphangiogenesis, which is a similar process to the one described above, plays an important role in cancer development. However, it is related to the lymphatic system. It consists in the growth of lymphatic vessels from the already existing ones [13]. It can be observed during wound healing, in inflammatory processes or in neoplastic disease. The development of a new vessel is complex and, as in the case of angiogenesis, consists of the same consecutive stages, all of which are strictly regulated by growth factors and selected proteins. 

Proangiogenic factors are divided into non-specific (e.g., EGF, FGF, PDGF) and specific factors (e.g., VEGF) [14]. Among the latter ones, there are three families of proteins, i.e., VEGF, ephrins and angiopoietins with their receptors. Ephrins with angiopoietin are mainly responsible for vascular maturation, whereas the VEGF family initiates angiogenesis [14]. The most important factors stimulating the processes of angio- and lymphangiogenesis include the vascular endothelial growth factor family. It consists of six structurally related proteins (VEGF-A, -B, -C, -D, PIGF, and VEGF-E) that regulate the growth and differentiation of many components of the vascular system, especially blood and lymphatic vessels [15]. The best-known proangiogenic factor is VEGF-A (formerly known as a “vascular permeability factor” [16]), whose gene is located on chromosome 6 (6p21.3) [17,18]. It is synthesized by many types of normal cells (ECs, vascular smooth muscle cells, monocytes, macrophages, mast cells, fibroblasts, keratinocytes, T lymphocytes, eosinophils) and by cancer cells and cancer-associated fibroblasts (CAFs) [19]. VEGF-A is responsible for increased permeability and vasodilatation by stimulating ECs to increased nitric oxide (NO) secretion [20]. This factor also stimulates proteolytic enzymes, thus having a significant effect on vascular remodeling. By binding to VEGFR-1 and -2 receptors with tyrosine kinase activity, VEGF-A stimulates ECs to proliferation, migration and protects them from apoptosis [9,21,22]. VEGF-A is the most important cytokine inducing angiogenesis [16,23]. The interaction of VEGF-A with its VEGFR-2 receptors located on the surface of ECs is mainly responsible for neoangiogenesis. On the other hand, by their binding to VEGF family proteins, VEGFR-1 receptors decrease the availability of the above proangiogenic factors for VEGFR-2 receptors, thus decreasing the intensity of angiogenesis induced by VEGFR-2 [14]. The VEGF-2 receptor plays a crucial role in both angiogenesis and lymphangiogenesis, which indicates that both processes are corelated. Other ligand and VEGF receptor variants are of secondary importance in angiogenesis [24,25,26,27]. In turn, VEGF-B, whose gene is located on chromosome 11 (11q13) [28], binds to the VEGFR-1 receptor, which results in tumor progression independently of angiogenesis [15]. 

During lymphangiogenesis, VEGF-C and -D factors play the main role, whereas VEGF-A has a supporting role. VEGF-C has its gene in chromosome 4 (4q34) [28]. This factor binds to the VEGFR-3 receptor, which leads to increased vascular permeability and stimulates migration and proliferation of ECs of lymphatic vessels. Moreover, it leads to the proliferation of lymphatic vessels in the skin [29,30]. The VEGF-D factor is another representative of this protein family. It is a product of gene expression located on the X chromosome (Xp21.1) [31]. As in the case of VEGF-C, this factor binds to the VEGFR-3 receptor, which stimulates the proliferation of ECs of lymphatic vessels. Moreover, studies have shown that increased expression of VEGF-D in epidermal cells leads to the formation of lymphatic vessels in the skin as in the case of VEGF-C [29,30]. Of note, VEGF-C and VEGF-D factors do not stimulate only lymphangiogenesis. By binding to both VEGFR-2 and VEGFR-3 receptors, they also participate in angiogenesis. A similar phenomenon is reported for VEGF-A, which is not only the main activator of angiogenesis, but it also stimulates lymphangiogenesis by the VEGFR-2 receptor. In turn, the *PIGF* gene, located on chromosome 14 (14q24-31) [18], encodes PIGF that binds to VEGFR-1 receptors and neuropilin-1 and is responsible for the stimulation of EC and smooth muscle cell growth. Together with VEGF-B, PIGF affects the differentiation and activation of monocytes. Furthermore, a significant increase in PIGF concentration in tissues was reported in myocardial infarction or cancer disease [29,30]. VEGF-E is the last of this group of proteins. It resembles the structure of VEGF-A in 25% [32] and is produced only by the orf virus. VEGF-E binds to its VEGFR-2 receptor, which results in EC proliferation and increased capillary permeability [29,30]. 

However, it should be borne in mind that ECs can be stimulated not only by the VEGF family of proteins, but also by various proangiogenic factors (Table 1), which can activate their proliferation and motility, leading to the initiation of angiogenesis when the synthesis of anti-angiogenic factors, such as angiostatin, endostatin, tissue inhibitors of metalloproteinases or retinoic acid [33,34], is reduced [33,35].

Some of the above endogenous inhibitors of angiogenesis are part of larger proteins, which do not independently regulate the formation of new blood vessels. These factors, including endostatin, can be released only under the influence of proteolytic enzymes such as elastases [14]. Endostatin blocks the activation of MAPK in ECs and MMPs, thus inhibiting angiogenesis [14]. The cis-4-proline hydroxylase enzyme also plays an important role, which under normoxia leads to the degradation of hypoxia-inducible factor 1α (HIF-1α) by oxygen attachment to proline residues in HIF-1α [14]. This allows the von Hippel-Lindau (VHL) protein to bind to HIF-1α and attach ubiquitin. This, in turn, leads to proteasomal degradation of HIF-1α protein and prevents angiogenesis by inhibiting *VEGF* gene expression [14]. Furthermore, many reports have indicated that periostin (POSTN), which is an ECM protein, may play a significant role in angiogenesis [39,40]. 

## 2. Periostin (POSTN)—Structure and Function

POSTN is an adhesion protein physiologically secreted by mesenchymal cells [41], which was first identified in the osteoblasts of MC3T3-E1 mice [40,42,43,44,45]. This protein is encoded by the *POSTN* gene [42] that is located in chromosome 13 (13q13.3) [43,46,47]. The *POSTN* gene contains 23 exons in humans [43,47]. 

**Figure 2:**
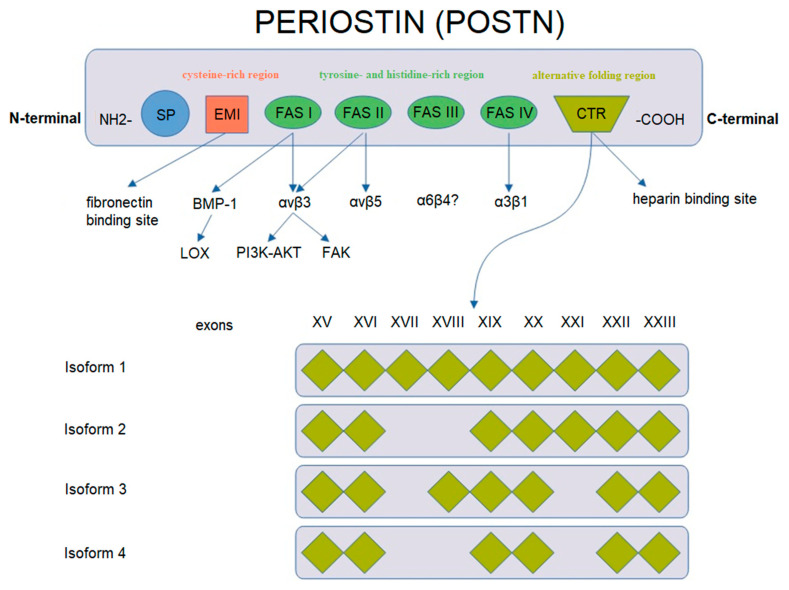
Schematic representation of *POSTN* gene structure and protein isoforms (based on Nuzzo et al. [43], modified).

POSTN has a characteristic structure (Figure 2). This glycoprotein is composed of several domains. It includes two terminal regions, i.e., N-terminal and C-terminal domains. The N-terminal region includes a signal peptide (SP), the cysteine-rich EMI domain and consecutive tandem repeats of four FAS1 domains. The FAS1 domain acts as a ligand of integrins of the cell membrane. In turn, the C-terminal region consists of a hydrophilic domain that regulates ECM organization and allows for interactions by binding ECM proteins such as type I and V collagen, fibronectin and glycosaminoglycans [43,44,48,49].

POSTN undergoes alternative folding in its C-terminal region, which leads to the formation of specific isoforms [48]. There are 4 isoforms of POSTN that consist of 751–836 amino acids with the molecular masses ranging from 83 to 93 kDa [43,50]. Isoform 1 is a full length variant with all exons. Isoform 2 lacks exons 17 and 18, isoform 3 lacks exons 17 and 21 and isoform 4 lacks exons 17, 18 and 21 [43]. The process of alternative folding of POSTN is generally understood [51,52], as opposed to the role of the isolated isoforms of the protein in the progression and metastasis of cancer cells.

Different isoforms have been identified in various tissues, i.e., isoform 1 in human osteosarcomas, isoform 2 in the placenta and isoform 3 in ovarian cancer, whereas isoforms 2 and 4 are found in normal tissues (breast, lung, thyroid, skin, ligaments, periosteum and periodontium [44,48,53,54,55,56,57,58,59]) and in bladder cancer [42,43,51,60,61].

**Figure 3:**
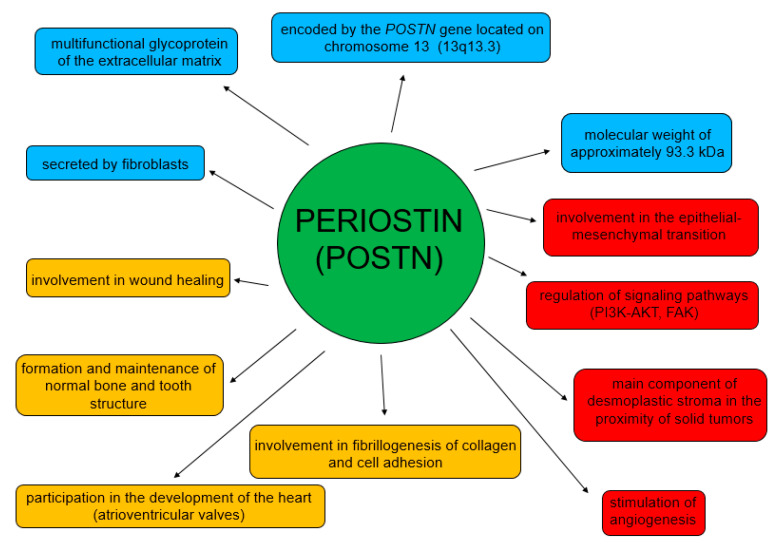
Physiological and pathological roles of POSTN.

POSTN participates in both physiological and pathological processes (Figure 3) [62]. Its physiological role is related to the process of wound healing [46,63]. It is also involved in the formation and maintenance of normal bone and tooth structure [44], and in the development of heart valves [64,65]. This glycoprotein is involved in myocardial remodeling after myocardial infarction and also in pulmonary vascular remodeling [66,67,68]. 

**Figure 4:**
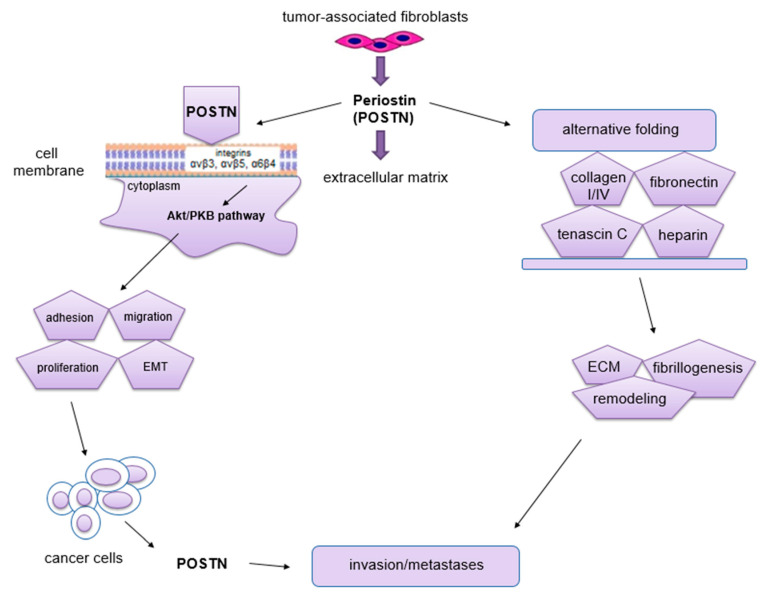
Schematic representation of the role of POSTN in carcinogenesis. This protein binds to integrins and activates the Akt/PKB and FAK signaling pathways, which results in tumor invasion and metastasis formation. POSTN isoforms bind ECM molecules, which affects the overall organization of the ECM (the figure is based on Morra et al. [48], modified).

In turn, the functions of POSTN in pathological processes are mainly related to its participation in migration, invasion, epithelial-mesenchymal transition (EMT) and metastasis formation [54,58,60,69,70] (Figure 4). Moreover, it stimulates tumor angio- and lymphangiogenesis [71].

The phenomenon of metastasis is closely related to the processes of migration and invasion of cells, both of which occur as a result of interactions between tumor cells, ECM and normal ECs [72]. The migratory potential of tumor cells depends on their functional state and the factors produced by the surrounding microenvironment that affect them [72]. Impaired angiogenesis is one of the most important factors crucial for metastasis formation [72]. It is known, that through its interaction with membrane integrins αvβ3, αvβ5 and α6β4 [60,73] and activation of Akt/PKB and FAK signaling pathways [69,73], POSTN affects cell migration. In addition, this protein can enhance cell migration by regulating collagen I fibrillogenesis in ECM [74]. Thus, this protein affects the biomechanical properties of the entire connective tissue. The changes that occur in the tumor microenvironment during this process can have a significant impact on increasing the invasiveness of tumor cells as well as their migration [48]. Baril et al. [73] showed that in pancreatic cancer, POSTN causes phosphorylation of FAK kinase by binding to integrin α6β4, which induces cell migration. In turn, cytokines such as TGF, IL-4 and IL-13 enhance the expression of POSTN, which interacts with integrin αvβ3, causing phosphorylation of Akt and FAK kinases, resulting in increased migration of cardiac myofibroblasts. POSTN with tenascin-C and fibronectin forms a tangled structure that is a specific scaffold for type I collagen [75]. In ovarian cancer, POSTN secreted by CAF cells after binding to the same integrin αvβ3 activates PI3K/Akt signaling pathways and the epithelial-mesenchymal transition (EMT) [76]. Through binding to integrin receptors (αvβ3, αvβ5, α6β4), POSTN activates signaling pathways (PI3K, AKT/PKB), which activate the ras-raf-MEK-MAPK pathway that initiates EMT [77]. All of the processes described above contribute to increased cell migration. Moreover, the ability of cancer cells to migrate is highly dependent on cell morphology, cell polarity, the presence of intercellular junctions and the expression of specific markers. Epithelial cells adhere closely to each other, forming intercellular junctions, in which E-cadherin (epithelial marker) is present. In addition, they have an apical-basal polarity, which results in the fact that these cells migrate poorly and are more likely to disseminate as cell conglomerates (collective migration). In turn, mesenchymal cells have significantly fewer intercellular junctions compared to epithelial cells. They are linked to the ECM by integrins located on their surface. Importantly, they also have the ability to secrete ECM-degrading enzymes. Mesenchymal cells penetrate relatively easily into capillary blood vessels at the site of the primary tumor, and hence their ability to migrate is high [78]. In the body, even mature cells possess some flexibility and the ability to change their phenotype. Physiologically, it occurs at the time of wound healing, whereas this process is best seen in cells after cancer transformation [72,79]. Epithelial cells can change their phenotype to a mesenchymal-like phenotype by EMT. It is a process in which the epithelial cell loses polarity and intercellular junctions and gains the ability for migration and invasiveness, thus becoming a cell with mesenchymal characteristics [80]. Moreover, this phenomenon is characterized by the loss of expression of markers such as E-cadherin, claudin, cytokeratin or desmoplakin that are characteristic of the epithelial phenotype. In turn, this process is characterized by increased expression of other factors, including fibronectin, N-cadherin, collagen, MMP-2, MMP-9 and MMP-15, which are typical of the mesenchymal phenotype [80,81]. EMT is a key mechanism in the processes of embryogenesis, regeneration, immune response and scar and fibrosis formation [82]. Moreover, this phenomenon is found in cancer development and progression. It also accompanies the formation of distant metastases [83], not only by facilitating the migration and invasion of tumor cells, but also causing their resistance to apoptosis due to the lack of adhesion [84]. Following metastasis, such cells are able to undergo a process opposite to EMT, which is known as the mesenchymal-epithelial transition (MET). 

EMT can be initiated by multiple mechanisms. Stimulated receptors of tyrosine and serine/threonine kinases (PI3K, EGFR, c-KIT) activate signaling pathways that trigger the ras-raf-MEK-MAPK pathway that initiates the EMT process [77]. It has been shown that due to its tyrosine- and histidine-rich regions, POSTN binds to integrin receptors (αvβ3, αvβ5, α6β4) [39,71] that are present on the surface of cancer cells, thus influencing the regulation of intracellular signaling pathways (PI3K, AKT/PKB) [69,71]. As a result, this protein plays an important role in the regulation of EMT, invasion and metastasis formation [48,69,73]. However, other studies showed that POSTN expression could be regulated by some growth factors, including TGF-β1 or BMP-2 [44,85]. It is believed that POSTN may act as a mediator to stimulate TGF-β1 to promote EMT and metastasis formation in some cancers [47].

As a protein with multiple functional domains, POSTN can interact with different proteins and may be involved in cancer progression. Therefore, this glycoprotein has become an interesting target for research in the context of cancer transformation and progression. Ma et al. [86] showed that POSTN, which is secreted by CAF cells activates FAK-Src kinases through its interactions with integrins (αvβ3, αvβ5), which leads to activation of the YAP/TAZ signaling pathway and an increase in YAP/TAZ proteins in the nucleus of cancer cells. In addition, Ma et al. provided strong evidence to support the hypothesis that the periostin-IL-6 loop contributes to regulating the interaction between tumor cells and fibroblasts during colorectal tumorigenesis. Targeting periostin- and IL-6- mediated tumor-stroma interaction may be an attractive therapeutic strategy for human colorectal tumors.

In addition, Yu et al. [87] showed that POSTN secreted by CAF cells in head and neck cancer could bind to the PTK7 kinase, which activates the signaling pathway mediated by the Dvl2 protein [88]. This, in turn, promotes phosphorylation of the GSK3β kinase and hypophosphorylation of β-catenin protein, which leads to the accumulation of β-catenin in the cytoplasm and allows it to enter the cell nucleus. The above factors indicate that the POSTN-PTK7 complex activates the Wnt signaling pathway [87]. In addition, it was demonstrated that POSTN promoted the cancer stem cell (CSC)-like phenotype via the PTK7-Wnt/β-Catenin signaling pathway [89,90] and enhanced proliferation and cell invasion in head and neck squamous cell carcinoma (HNSCC) [87].

In turn, Kubo et al. [91] showed that proteins such as POSTN, tenascin-C (TNC) and fibronectin (FN) interacted and formed the ECM protein complex promoting angiogenesis in patients with ischemic proliferative retinopathy. In addition, POSTN has been shown to facilitate incorporation of TNC into the ECM complex [91]. This is supported by previous findings where it was suggested that through its domains, POSTN bound to TNC and other ECM proteins, which enabled the connection between TNC and ECM [92]. In addition, a significant correlation was demonstrated between the protein levels (POSTN, TNC and FN) in the vitreous body of patients with proliferative diabetic retinopathy and their distribution in newly formed vessels in the retina. In addition, Th2 cells infiltrating the ischemic retina can secrete IL-13. This cytokine stimulates EC cells, enhancing the secretion of POSTN, TNC and FN proteins, which stimulates angiogenesis in the retina in patients with ischemic proliferative retinopathy [91].

## 3. The Role of POSTN in Angiogenesis and Lymphangiogenesis

POSTN is involved in the formation of new blood vessels during tumor transformation [71]. Initially, when the tumor is small, tumor cells obtain oxygen and nutrients from the blood by diffusion. When its volume exceeds about 2 mm^3^, obtaining essential substances in this manner is insufficient [10,93,94]. The growing environment becomes hypoxic and acidified due to excess metabolic products [10]. Baril et al. [73] showed that high POSTN expression made cancer cells resistant to hypoxia [73]. Moreover, these cells with normal cells stimulate angiogenesis [10,93] via pathways (FI3K-AKT, FAK, Erk/VEGF) [71,95]. The PI3K/AKT pathway is initiated by ligand attachment to a tyrosine kinase receptor in tumor cells, which results in PI3K activation. In turn, this kinase converts phosphatidylinositol 4,5-bisphosphate (PIP2) to phosphatidylinositol 3,4,5-triphosphate (PIP3), which binds AKT kinase to the cell membrane where it is activated. This results in the inhibition of apoptosis, increased proliferative activity and cell migration potential [96,97,98]. However, in the FAK pathway, the cytoplasmic tyrosine kinase is mediated in signal transduction to intracellular proteins. POSTN can interact with the above pathway by increasing the expression of the VEGF-R2 receptor in endothelial cells via the αvβ3 integrin [99], thus having an effect on regulating angiogenesis. In turn, the Erk/VEGF pathway is induced by binding of the growth factor VEGF-A to the VEGF-R2 receptor, which results in phosphorylation of phospholipase C gamma (PLC-γ) and activation of MAPK/Erk signaling pathways [100,101]. It was shown that increased POSTN expression enhanced VEGF expression and promoted the Erk phosphorylation by forming new vessels and metastases [95].

POSTN is a protein secreted by both tumor cells [69,71,102] and cancer-associated fibroblasts (CAFs) [39,40,71,102,103]. This glycoprotein is thought to influence the development of new blood vessels by regulating two mechanisms, i.e., from ECs of an already existing vessel and the recruitment of progenitor cells [104,105,106]. POSTN enhances the adhesion and migration of Ecs by interacting with the αvβ3 integrin [40,47,48]. Moreover, high expression of this integrin was shown in Ecs during inflammation or interaction of growth factors with Ecs [12]. The relationship between POSTN expression and VEGF proangiogenic factors is well reported in many cancers [99,107]. However, in the case of non-small cell lung carcinoma (NSCLC), a similar correlation has been analyzed only by a small number of researchers [108]. This issue is relatively well understood in terms of breast cancer. Shao et al. [99] demonstrated that increased POSTN expression was correlated with increased VEGF receptor (Flk-1/KDR) expression, which stimulates angiogenesis in breast cancer. Similarly, Puglisi et al. [41] demonstrated a correlation between POSTN expression and VEGF-A, VEGF-R1 and VEGF-R2 receptors, and suggested that POSTN could be important in angiogenesis in breast cancer. On the other hand, in their study on oral squamous cell carcinoma (OSCC), Siriwardena et al. [109] found a significant correlation between POSTN expression and microvessel density (MVD) in tumors with high POSTN expression as compared to tumors with low expression of the glycoprotein. Furthermore, the effect of recombinant POSTN (rPOSTN) on the formation of new capillaries was demonstrated in an in vitro model [109]. 

POSTN is also thought to play an important role in lymphangiogenesis, which is crucial for the formation of metastases, which translates into cancer progression. The lymphatic system plays a key role in maintaining adequate fluid amount in tissues, lipid absorption, and immune cell transport. Congenital or acquired failure of these vessels results in various forms of lymphedema [110,111]. POSTN expression was reported to influence the formation of new lymphatic vessels in tumors [112]. Their development and the involvement of lymph nodes by tumor cells indicate disease progression [113,114]. The VEGF-C/VEGFR-3 system is the most important regulatory mechanism responsible for the development of these vessels. Jeltsch et al. [115] and Anisimov et al. [116] demonstrated that increased expression of VEGF-C in different tissues led to lymphatic vessel expansion. In turn, He et al. [117] showed that blocking VEGFR-3 receptor signaling using VEGFR-3 immunoglobulin (VEGFR-3-Ig) could lead to the inhibition of lymphangiogenesis and reduced metastasis to the surrounding lymph nodes in lung cancer [117]. To achieve it, two stable cell lines were obtained, i.e., N15 with low metastatic capacity secreting VEGF-C and the highly metastatic LNM35 cell line synthesizing VEGFR-3-Ig. These lines were implanted subcutaneously into immunodeficient mice. The expression levels of VGFR-C in tumor cells and the number of lymphatic vessels in tumors were assessed [117]. Kudo et al. [112] showed a significant correlation between POSTN and VEGF-C expression. Moreover, it was demonstrated that POSTN expression alone could induce lymphangiogenesis in head and neck squamous cell carcinoma (HNSCC) through the activation of intracellular Src and Akt [112]. In turn, in their study on NSCLC, Takanami et al. [108] showed a significant association between POSTN expression and lymphatic vessel density (LMVD) assessed based on podoplanin (D2-40) expression and suggested that increased POSTN expression enhanced lymphangiogenesis. 

POSTN is not the only ECM protein that has the ability to regulate tumor angiogenesis. Proteoglycans (e.g., perlecan, syndecan and agrin) that are part of the ECM are also involved in the formation of new blood vessels. Similar to POSTN, agrin, perlecan and syndecan affect the process of angiogenesis through interactions with the most important growth factor (VEGF), causing activation of the VEGF-VEGFR2 pathway [99,118,119,120].

Agrin facilitates the binding of VEGF to the VEGFR2 receptor [121]. Similarly, the N-terminal domain of perlecan facilitates the binding of VEGFA and FGF to their receptors [118]. A similar mechanism is demonstrated by syndecans (SDCs), which act as coreceptors for VEGF [122]. They bind to VEGF by increasing their concentration in the cell membrane, facilitating their binding to their VEGFRs [122]. In turn, an increase in POSTN expression enhances VEGF expression and promotes Erk phosphorylation and hence POSTN enhances angiogenesis [95]. Interestingly, a decrease in SDC2 expression results in a decrease in phosphorylated Src and phosphorylated ERK, which inhibits the KRas/MAPK pathway, and thus SDC2 indirectly affects angiogenesis [123]. Studies have shown that by increasing VEGF-R2 receptor expression via integrin αvβ3, POSTN regulates the FAK signaling pathway [99], which is also activated by the agrin receptor complex consisting of integrin β1, Lrp4 and MuSK [121]. Through the FAK signaling pathway, POSTN and agrin can influence angiogenesis [99,121]. The same is true when SDC1 binds to IGF1-R and undergoes autophosphorylation and activation. This results in the activation of talin, which, in turn, activates integrins αvβ3 and αvβ5 and activates signaling pathways that can affect angiogenesis [123,124,125]. In turn, SDC2 interacts with the protein tyrosine phosphatase receptor CD148 which activates the PI3K pathway that regulates angiogenesis [126]. Similar to SDC2, POSTN uses the PI3K/AKT pathway to affect angiogenesis through this mechanism [69].

## 4. Effect of POSTN Expression on Angiogenesis and Metastasis

**Figure 5:**
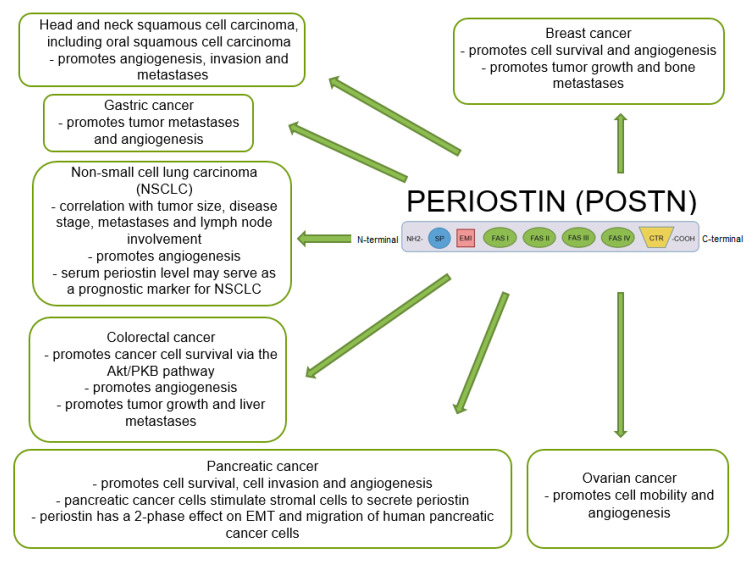
Schematic representation of the role of POSTN expression in various cancers (based on Ruan et al. [47], modified).

Many studies indicate that POSTN plays an important role in tumor growth and the formation of new blood and lymphatic vessels in its proximity. A significant positive correlation was shown between the increase in POSTN expression and metastases in various cancers [47,48,69,109,127,128,129,130], including NSCLC, breast, ovarian, pancreatic or colorectal cancer [60,131] (Figure 5).

It has been also shown that POSTN may be a diagnostic biomarker. Jia et al. [132] showed that serum POSTN was a promising potential biomarker for the diagnosis and prediction of metastases in breast cancer, supporting diagnoses based on CA153 and CEA antigens. Similarly, a study by Rachner et al. [133] showed that POSTN serum levels in breast cancer patients could be a potential biomarker in predicting disease progression, regardless of the presence of metastases. In addition, the higher the POSTN serum levels in patients, the higher the mortality rate compared to the group with low levels of the glycoprotein. In turn, Ben et al. [134] showed that POSTN serum levels in patients with colorectal cancer were significantly higher compared to healthy individuals and patients with benign tumors. In addition, a correlation was observed between higher preoperative serum POSTN levels in colorectal cancer patients and the formation of distant metastases or a poor prognosis in patients. 

Xu et al. [135] showed that POSTN serum levels were significantly elevated in patients with non-small cell lung cancer compared to healthy individuals. Overall survival and disease-free survival were significantly higher in patients with lower POSTN serum levels compared to the group with higher POSTN levels. It is suggested that POSTN serum levels can be considered diagnostic and prognostic markers in patients with non-small cell lung cancer.

### 4.1. Breast Cancer

Many reports have suggested that increased POSTN expression significantly enhances angiogenesis in breast cancer. Försti et al. [136] demonstrated the involvement of VEGF through the kinase domain receptor (KDR). VEGF was the main inducer of angiogenesis in breast cancer. An increase in POSTN expression stimulates this receptor, which initiates angiogenesis [136]. In turn, Puglisi et al. [41] found a significant positive correlation between POSTN expression, which was localized in the nuclei of breast cancer cells, and tumor size and the expression of VEGF-A, VEGF-R1 and VEGF-R2 receptors [41], which suggests an important role of POSTN in angiogenesis of this tumor. Similar results were also obtained by Shao et al. [99], who demonstrated a phenotype of accelerated breast cancer growth and enhanced angiogenesis [99] in xenografts in immunocompromised mice which were injected with cell lines (293T, B16F1, MDA-MB-231) overexpressing POSTN. Furthermore, it was found that POSTN enhanced the expression of the VEGF-R2 receptor in ECs through the activation of the αvβ3-FAK signaling pathway [99], which may significantly affect new blood vessel formation. In turn, Lee et al. [137] showed that benzyl-d(U)TP-modified DNA aptamer that was directed against human POSTN selectively bound to FAS-1 domain (PNDA-3) of POSTN inhibited breast cancer growth and metastasis in vivo. In an orthotopic mouse model with silenced POSTN expression by PNDA-3, reduced primary tumor growth and distant metastasis were found. Although it effectively inhibited tumor growth in vivo, PAND-3 had a poor effect on cell growth in vitro. This difference can be explained by the fact that POSTN does not only affect tumor cells but it also influences the cells surrounding the tumor, such as endothelial cells. POSTN can induce angiogenesis by binding to integrins αvβ3 and αvβ5 and activating the integrin-FAK signaling pathway. In that study, the authors determined the anti-angiogenic effect of PNDA-3 both in vitro and in vivo models. In vitro studies and the assessment of tumor sections from PNDA-3-treated mice showed reduced angiogenesis compared to both the vehicle and control aptamer-treated groups. PNDA-3 was shown to strongly inhibit adhesion, migration and invasion of breast cancer cells. 

### 4.2. Ovarian Cancer

POSTN expression significantly enhances angiogenesis and metastasis in ovarian cancer [138]. This phenomenon significantly contributes to a poorer prognosis and makes this cancer the most lethal gynecological malignancy (2019) [139]. In an in vitro study of ovarian cancer cell lines (OVCAR-3 and OV2008) with POSTN overexpression obtained by retroviral transfection, Zhu et al. [138] showed that POSTN did not affect the proliferative activity of tumor cells. However, this glycoprotein enhances migration and adhesion of human umbilical vein endothelial cells (HUVEC) by interacting with integrin receptors (αvβ3, αvβ5). This mechanism stimulates angiogenesis in tumors and may inhibit apoptosis of ovarian cancer cells. Furthermore, in vivo studies showed that this protein promoted metastases of intraperitoneally implanted tumor in immunosuppressed mice [138]. Microvessel density (MVD) of tumor xenografts in mice was assessed by CD31 staining. Tumor cell xenografts overexpressing POSTN showed higher MVD values compared to control cells [138], which suggests the involvement of POSTN in blood vessel formation and association with a phenotype of increased tumor angiogenesis and decreased tumor cell apoptosis. 

### 4.3. Pancreatic Cancer

Many studies have suggested that the process of angiogenesis also plays an important role in pancreatic cancer. Liu et al. [95] demonstrated that the proliferative activity, migration and invasive potential of endothelial cells (HUVECs) were significantly increased in the cell line cultured with recombinant POSTN (rPOSTN) compared to the control line where this protein was added. In vivo studies showed that reduced POSTN expression in pancreatic cancer cells inhibited tumor growth and significantly decreased VEGF expression in mice, which resulted in a decrease in the number of metastases and inhibited angiogenesis. The formation of new blood vessels was closely correlated with metastasis formation and a poor prognosis. These authors suggested that POSTN promoted angiogenesis in pancreatic cancer by activating the Erk/VEGF signaling and it could become a therapeutic target in this cancer [95].

Many reports have shown that traditional anti-angiogenic drugs are ineffective and may even enhance tumor progression. By inducing hypoxia, these substances can paradoxically stimulate the process of angiogenesis, which can cause metastasis [95].

Molecular and cellular mechanisms underlying tumor resistance to VEGFA neutralization are diverse and not fully understood. Keklikoglou et al. [140] showed that in a mouse model of pancreatic neuroendocrine tumor (PNET), de novo POSTN deposition adapted the tumor to chronic inhibition of VEGFA by sustaining macrophage infiltration. The process of angiogenesis and PNET progression when VEGFA protein is inhibited is POSTN-dependent. Genetic deletion of POSTN in RIP1-Tag2 mice blunted tumor rebounds of M2-like macrophages and αSMA+ stromal cells in response to prolonged VEGFA inhibition. This leads to inhibition of revascularization. POSTN deficiency also inhibits FGF2, which is an adaptive mechanism enabling effective anti-angiogenic therapy in PNET. These results indicate that POSTN plays an important role in resistance to anti-VEGFA therapy in pancreatic neuroendocrine tumor (PNET).

Many reports suggest that most of the pancreatic cancers are directly invasive to the surrounding tissues independent of their poor tumor vasculature, thus, pancreatic cancer is generally resistant to anti-angiogenic therapy such as anti-VEGF antibody.

The results Carbone et al. [141] are in support of the hypothesis that actually the resistance to anti-VEGF therapy is mediated by tumor cells autonomous secretion of chemokines that have both paracrine and autocrine effect [142]. They showed that gene expression profiles of bevacizumab resistant model highlighted an increased production of chemokines that are important for the attraction of myeloid cells and mobilization of their precursors from bone marrow. These cells have a role both in promotion of tumor angiogenesis [143] as well as in maintenance of an inflammatory environment that sustains tumor progression [144]. These observations can explain the lack of efficacy of anti-VEGF agents since the positive effect of blood supply reduction is counteracted by indirect pro-angiogenic and pro-inflammatory effect [142].

### 4.4. Non-Small Cell Lung Carcinoma (NSCLC)

The role of POSTN expression in the process of angiogenesis has also been analyzed in NSCLC. However, this phenomenon has been discussed only in several papers [108,145]. Wu et al. [145] reported that high POSTN expression in tumor cells correlated with increased formation of new blood vessels and metastasis formation in NSCLC. In addition, this protein significantly affected proliferation and invasion of cancer cells and EMT. Their study revealed that tumor cells (A549) with silenced POSTN expression had significantly reduced expression levels of Snail protein, which was responsible for EMT induction [145]. Moreover, their study demonstrated that this glycoprotein could be an independent prognostic factor and even a potential therapeutic target in NSCLC [145]. 

In turn, Takanami et al. [108] demonstrated a significant correlation between POSTN expression in the cytoplasm of tumor cells and new lymph vessel formation [108], tumor size [102,108], invasion and lymph node involvement. Furthermore, a positive correlation was found between POSTN expression and LMVD [108]. Furthermore, Takanami et al. [108] showed a similar correlation to the one shown by Siriwardena et al. [109] between POSTN expression and estimated MVD in NSCLC based on the expression of von Willebrand factor-related antigen (F8RA). In addition, Takanami et al. [108] demonstrated a positive significant correlation between POSTN expression and clinicopathological factors and patient survival time. Five-year survival rates were significantly higher in patients with no POSTN expression in tumor cells compared to subjects with the expression of the glycoprotein [108]. Of note, their study was based on only 88 cases of NSCLC, with only 42% of them showing positive POSTN expression [108]. 

### 4.5. Colorectal Cancer (CRC)

Studies have confirmed the effect of POSTN expression on new blood vessel formation in CRC. Bao et al. [69] demonstrated a significant positive correlation between high POSTN expression and the presence of liver metastases and stimulation of angiogenesis via the Akt/PKB pathway [69]. Their results showed that the protein could play an important role in new vessel formation and metastasis formation in CRC. The increased expression of POSTN in CRC was also confirmed (80% of the cases) as compared to healthy intestinal tissues from the tumor margin, which may indicate an important role of the glycoprotein in tumor transformation. 

### 4.6. Glioblastoma Stem Cells (GSCs) 

The process of angiogenesis in terms of POSTN expression has also been evaluated in glioblastoma stem cells (GSCs) [146,147]. Huizer et al. as well as Zhou et al. [90,146] confirmed that pericytes and GSCs expressed POSTN, which recruited tumor-associated macrophages (TAMs) from the peripheral blood to the tumor proximity through integrin αvβ3-mediated signaling [90]. Through this mechanism, POSTN enhanced tumor progression and angiogenesis [146,147]. Moreover, it promoted migration and invasion of macrophages and monocytes to GSCs [90]. Zhou et al. [90] demonstrated that silencing POSTN expression in GSCs impaired the recruitment of TAMs, inhibited tumor growth, and increased survival in mice with transplanted GSCs [90]. Additionally, Ouanouki et al. [148] demonstrated that silencing POSTN expression inhibited glioma cell invasion (U-87) through decreased expression of fibronectin, vimentin and reduced phosphorylation of Smad2, AKT and FAK in tumor cells. A similar relationship was shown by Mikheei et al. [149] who reported a significant relationship between POSTN expression and tumor stage and tumor recurrence after treatment. However, such a relationship was not found in relation to patient survival time. In addition, Tian et al. [150] demonstrated that POSTN could be an independent prognostic factor in GSCs. 

### 4.7. Gastric Cancer (GC)

The relationship between POSTN expression and angiogenesis has also been analyzed in GC. Qiu et al. [151] demonstrated that silencing of POSTN expression by interfering RNA (siRNA) in hypoxic (2% O_2_) gastric cancer cells (MKN-45) correlated with significantly reduced VEGF expression at the mRNA level, which indicated that POSTN was involved in the regulation of the pro-angiogenic factor VEGF. This affected the inhibition of new blood vessel formation by decreasing interaction of POSTN with the Erk1/2 signaling pathway mediated by VEGF [151]. 

### 4.8. Hepatocellular Carcinoma (HCC)

To date, there have been few studies on POSTN expression in HCC [73,152,153]. Chen et al. [154] identified POSTN as an effector protein in sulfatase 2 (SULF2)-induced angiogenesis in HCC. In vivo studies showed that silencing the expression of the protein in tumor cells inhibited new blood vessel formation and significantly suppressed tumor growth in mice. Additionally, Chen et al. [154] identified a signaling pathway (TGFβ1/SMAD) that allowed the interaction between SULF2 and POSTN expression. Furthermore, they suggested that the SULF2/POSTN relationship could become a target for the development of new therapies in HCC [154]. In turn, Lv et al. [155] indicated a significant correlation between the expression of POSTN and VEGF in HCC. Tumors showing high POSTN expression were characterized by higher VEGF expression and higher MVD compared to those that showed no POSTN expression [155]. A similar relationship was analyzed by Lv et al. [155] and Jang et al. [156] who also demonstrated that high POSTN expression was associated with increased cancer disease, advanced disease stage and a poor prognosis. 

### 4.9. Head and Neck Squamous Cell Carcinoma (HNSCC) 

In head and neck squamous cell carcinoma (HNSCC), increased POSTN expression was closely correlated with increased tumor angio- and lymphangiogenesis. Kudo et al. [112] demonstrated a significant positive correlation between high expression of the protein and increased expression of VEGF-C in HNSCC cells, which promoted lymphangiogenesis through the activation of Akt and Scr signaling pathways. The Scr pathway was activated by binding of VEGF-C to VEGFR-2 and VEGFR-3 receptors [19,157,158], which induced lymphangiogenesis. Of note, VEGF-C did not only stimulate the formation of lymphatic vessels by binding to VEGFR-2 and VEGFR-3 receptors, but also participated in angiogenesis. In addition, using ELISA, these authors demonstrated a positive correlation between the expression level of POSTN and VEGF-C in the serum of HNSCC patients. In conclusion, POSTN, as well as VEGF-C protein, may affect the process of lymphangiogenesis and angiogenesis in HNSCC. 

### 4.10. Esophageal Squamous Cell Carcinoma (ESCC) 

In terms of angiogenesis, POSTN expression has also been analyzed in ESCC. Wang et al. [104] demonstrated that increased POSTN expression in ESCC significantly correlated with lymph node metastasis, tumor differentiation, tumor cell infiltration in venous vessels and the TNM staging. Moreover, it was associated with tumor progression, an increase in VEGF expression and angiogenesis [104]. Tumors with high POSTN expression were characterized by a higher expression level of VEGF located in the cytoplasm of tumor cells and a higher MVD compared with tumors with low expression of the glycoprotein. These results indicate that POSTN may play a key role in angiogenesis in ESCC. 

## 5. Conclusions

POSTN is a multifunctional ECM glycoprotein secreted by tumor cells and CAFs. It is a major component of the desmoplastic stroma that is formed in the proximity of solid tumors. It is involved in collagen fibrillogenesis [74] and cell adhesion [39,59]. It binds to integrin receptors (αvβ3, αvβ5, α6β4) [39,71] that are present on the surface of tumor cells and vascular endothelium, which allows regulation of signaling pathways (PI3K-AKT, FAK) [69,71]. Thus, this protein may have an effect on cell proliferation, tumor growth and the formation of new blood and lymphatic vessels [71], which may be of crucial importance in the mechanism of metastasis and cancer progression. 

## Figures and Tables

**Table 1:** Functions of selected proangiogenic factors.

Non-VEGF Proangiogenic Factors	Functions of Selected Proangiogenic Factors:
Class 3 semaphores (SEMA3)	modulate adhesion, migration, proliferation and apoptosis of ECs and alter vascular permeability [36].
Nogo-A	regulates EC migration and proliferation [37]. changes vascular permeability [38].
Fibroblast growth factor-2 (FGF-2)	promotes proliferation and differentiation of ECs, smooth muscle cells and fibroblasts [9].
Placental growth factor (PIGF)	increases vasodilation [9].
Angiopoietin 1 (Ang 1)	stimulates remodeling, maturation and stabilization of vessels [9].
Membrane metalloproteinases (MMPs)	play an essential role in the remodeling of the extracellular matrix [9].
Urokinase/tissue-type plasminogen activator (uPA/tPA)	initiates degradation of the extracellular matrix [9].
Monocyte chemoattractant protein-1 (MCP-1)	regulates dilation of collateral vessels [9].

## Data Availability

Not applicable.
